# Feasibility and accuracy of a novel automated three-dimensional ultrasonographic analysis system for abdominal aortic aneurysm: comparison with two-dimensional ultrasonography and computed tomography

**DOI:** 10.1186/s12947-020-00207-0

**Published:** 2020-07-01

**Authors:** In-Jeong Cho, Jinyong Lee, Jinki Park, Sang-Eun Lee, Chul-Min Ahn, Young-Guk Ko, Donghoon Choi, Hyuk-Jae Chang

**Affiliations:** 1 Division of Cardiology, Department of Internal Medicine, Ewha Womans University Seoul Hospital, College of Medicine, Ewha Womans University, Seoul, Republic of Korea; 2 Ultrasound R&D Group, Samsung Medison Co., Ltd, Seoul, Republic of Korea; 3 Division of Cardiology, Severance Cardiovascular Hospital, Yonsei University College of Medicine, 50 Yonsei-ro, Seodaemun-gu, Seoul, 03722 Republic of Korea

**Keywords:** Three-dimensional imaging, Abdominal aortic aneurysm, Software validation

## Abstract

**Background:**

Accurate measurement of the maximum aortic diameter (Dmax) is crucial for patients with abdominal aortic aneurysm (AAA). Aortic computed tomography (CT) provides accurate Dmax values by three-dimensional (3-D) reconstruction but may cause nephrotoxicity because of contrast use and radiation hazard. We aimed to evaluate the accuracy of a novel semi-automated 3-D ultrasonography (3-D US) system compared with that of CT as a reference.

**Methods:**

Patients with AAA (*n* = 59) or individuals with normal aorta (*n* = 18) were prospectively recruited in an outpatient setting. Two-dimensional ultrasonography (2-D US) and 3-D US images were acquired with a single-sweep volumetric transducer. The analysis was performed offline with a software. Dmax and the vessel area of the Dmax slice were measured with 2-D US, 3-D US, and CT. The lumen and thrombus areas of the Dmax slice were also measured in 40 patients with intraluminal thrombus. Vessel and thrombus volumes were measured using 3-D US and CT.

**Results:**

The Dmax values from 3-D US demonstrated better agreement (*R*^2^ = 0.984) with the CT values than with the 2-D US values (*R*^2^ = 0.938). Overall, 2-D US underestimated Dmax compared with 3-D US (32.3 ± 12.1 mm vs. 35.1 ± 12.0 mm). The Bland-Altman analysis of the 3-D US values, revealed better agreement with the CT values (2 standard deviations [SD], 2.9 mm) than with the 2-D US values (2 SD, 5.4 mm). The vessel, lumen, and thrombus areas all demonstrated better agreement with CT than with 2-D US (*R*^2^ = 0.986 vs. 0.960 for the vessel, *R*^2^ = 0.891 vs. 0.837 for the lumen, and *R*^2^ = 0.977 vs. 0.872 for the thrombus). The thrombus volume assessed with 3-D US showed good correlation with the CT value (*R*^2^ = 0.981 and 2 SD in the Bland-Altman analysis: 13.6 cm^3^).

**Conclusions:**

Our novel semi-automated 3-D US analysis system provides more accurate Dmax values than 2-D US and provides precise volumetric data, which were not evaluable with 2-D US. The application of the semi-automated 3-D US analysis system in abdominal aorta assessment is easy and accurate.

## Introduction

The European Society for Vascular Surgery defined abdominal aortic aneurysm (AAA) as an abdominal aortic diameter of ≥3.0 cm in either the anteroposterior or transverse plane [1]. AAA can be a life-threatening disease, and its incidence increases with age [2, 3]. However, even large aortic aneurysms rarely cause symptoms, [4, 5] and the risk of rupture is higher with increased maximum diameter (Dmax) and expansion rate [6–8]. Thus, AAAs require regular monitoring of Dmax and preventive surgery, and endovascular repair is proposed when an AAA reaches a Dmax of 55 mm or grows rapidly over 1 cm/year [1]. This makes accurate Dmax measurement an essential part of AAA management for diagnosis, follow-up before treatment, and planning for repair.

The most commonly used imaging technique for measuring AAA size is two-dimensional ultrasonography (2-D US), closely followed by computed tomography (CT) [9]. 2-D US is the mainstay imaging for screening and monitoring the growth of small AAAs because of its wide availability, painlessness, and low cost. However, it has limitations. It is user dependent and shows high interobserver variability and low reproducibility, [10] which are characteristics critical in serial surveillance. CT can measure Dmax perpendicular to the centerline of flow and is a useful tool to measure accurate AAA size with high reproducibility. However, it has fundamental limitations, including radiation exposure and use of ionizing contrast that causes renal toxicity. Since the report that 25–30% of patients with AAA had chronic renal failure, [11] the use of CT as a surveillance tool with repeated measurement has been limited.

To overcome the limitations of 2-D US and CT, several researchers have published investigations on novel methods for the analysis of three-dimensional (3-D), complex AAA shapes [12–14]. Among the various analytical methods, 3-D ultrasonography (3-D US) is an inexpensive and noninvasive method to assess the geometry of AAA without biological hazards. We developed a semi-automatic 3-D US software system that can automatically extract Dmax in any direction, perpendicular to the centerline, and allows calculation of the volumetric parameter of AAA. The aim of the present study was to assess the feasibility and accuracy of this novel automated 3-D US analysis system for AAA. We assessed the accuracy of this novel 3-D US analysis system in measuring Dmax and volumetric parameters in various sizes of the aorta and the accuracy of the values obtained compared with those obtained with CT as a gold standard.

## Methods

### Study population

We prospectively recruited 59 consecutive patients who had been diagnosed with AAA (Dmax ≥3 cm on aorta CT or 2-D US) at a single tertiary medical center between April 2016 and May 2017. The interval between aorta CT and ultrasonography was < 1 month (median, 13 days; interquartile range [IQR], 7–25 days), and all the patients underwent 2-D and 3-D US on the same day. The exclusion criteria were an estimated glomerular filtration rate of < 50 mL/min/1.73 m^2^. The control subjects were screened from among those who underwent abdominal contrast CT for clinical reasons. Eighteen subjects who agreed to participate were included and underwent 2-D and 3-D US within 6 months after the index CT (interval: median, 96 days; IQR, 92–98 days). Overall, 59 patients with AAA and 18 control subjects were enrolled in the final analysis of 2-D US, 3-D US, and CT. This study was approved by the institutional review board of Yonsei University, Severance Hospital, Seoul, Korea. Informed consent was obtained from all the patients.

### Analyzed vessel selection

In patients with AAA, the range of imaging for AAA assessment was selected on the basis of the Dmax slice. The images of the aortic wall within 20 mm cranially and 20 mm caudally from the Dmax slice were selected for analysis (total length, 40 mm). For the control subjects, the analyzed part was selected on the basis of the left renal artery orifice. All CT and ultrasonographic variables were calculated at the site that was 10 mm lower to the left renal artery orifice, with a length of 12 mm, in the control subjects.

### CT analysis

**Fig. 1:**
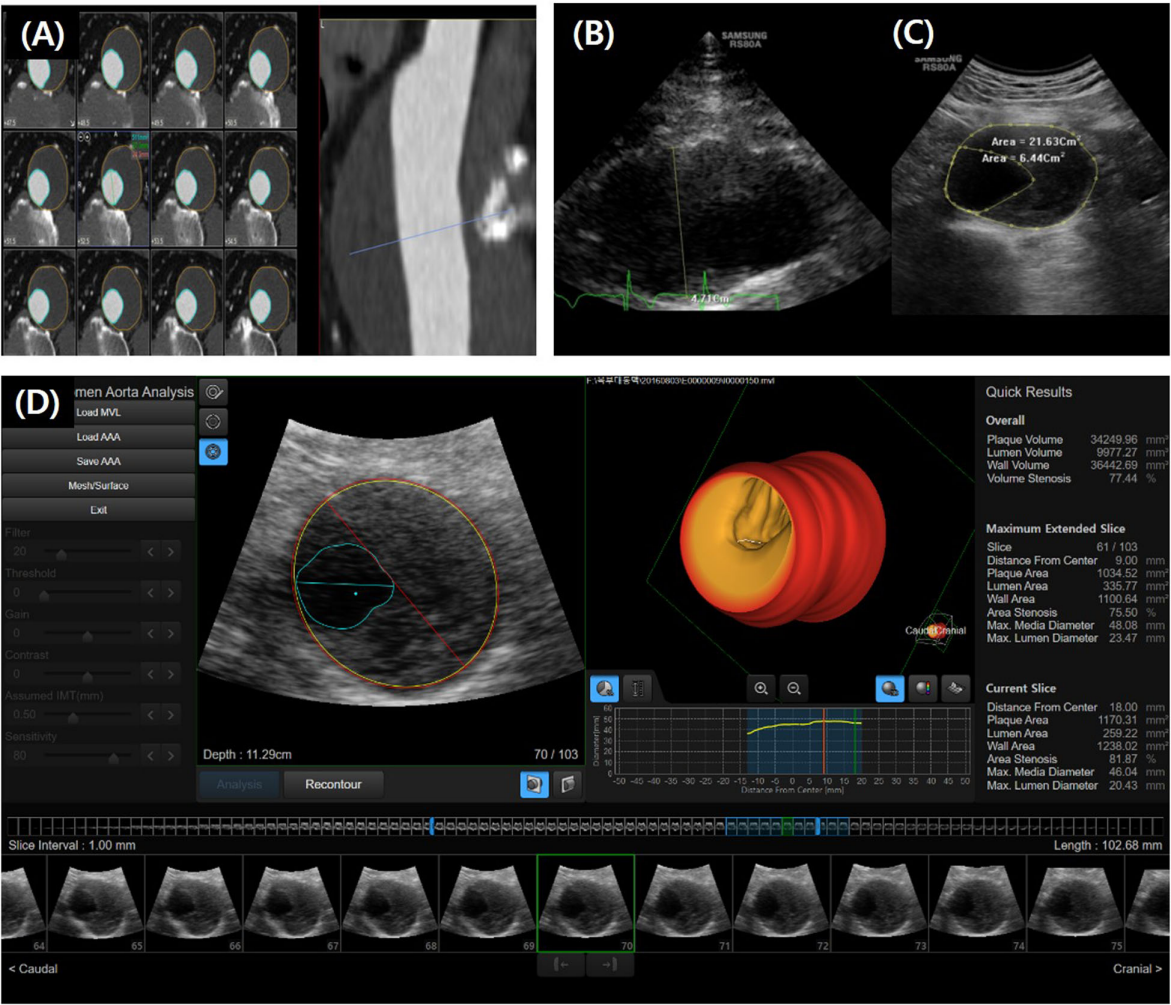
A representative case comparing the analysis of aortic diameter, area, and thrombus volume by different tests in abdominal aortic aneurysm: (**a**) aorta computed tomography, (**b**) two-dimensional (2-D) longitudinal, (**c**) 2-D axial images, and (**d**) three-dimensional (3-D) automated software analysis. The yellow circle marks the intima-lumen boundary; the red circle, the media-adventitia interface; and blue circle, the thrombus boundary in the automated 3-D ultrasonographic software analysis in D. The red line represents the maximum diameter of the vessel, and the blue line represents the maximum diameter of the non-thrombosed lumen

**Fig. 2:**
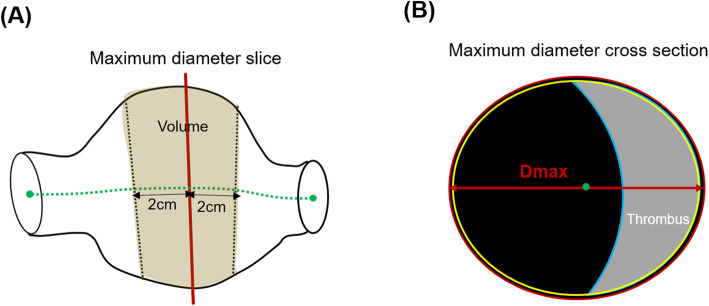
Schematic representation of the measurements: (**a**) maximum diameter cross-section. (**b**) Volume measurements. The lumen area and thrombus are defined as the maximum diameter (Dmax) cross-section. The vessel area is defined as the area surrounded by the red line, including the lumen and thrombus in the maximum diameter cross-section. The vessel volume is centered at the cross-section containing the Dmax and was limited by the cranial and caudal cross sections by 20 mm. The lumen and thrombus volumes were the volumes of the lumen and thrombus within the vessel, respectively

CT was used as the gold standard when US and CT were compared, and the entire aneurysm was displayed. The patients were scanned using a 64-section CT scanner (Sensation 64; Siemens Healthcare, Forchheim, Germany). Contrast-enhanced CT was performed using 100–140 kV with 150–220 mAs, depending on the patient’s size without electrocardiography (EGC) gating. Images were reconstructed with a slice thickness of 1.0 mm. The CT images were post-processed offline using dedicated software (Vitrea 2.0; Vital Images, Minnetonka, MN, USA; Fig. 1a). Dmax was measured perpendicular to the flow line of the vessel on 3-D reconstructed CT images [15]. The vessel, lumen, and thrombus areas were measured at the Dmax slice. The vessel volume at 40 mm was calculated with the Dmax slice located in the center of the volume. The lumen and thrombus volumes were also calculated within the same range of volume data for vessel volume measurement in the subgroup of patients with an intraluminal thrombus. A schematic representation of the measurement variables is shown in Fig. 2.

### US acquisition

The patients did not routinely undergo any specific preparations such as fasting before the US examination. After 10 min of rest, the patients were placed in the supine position. All 2-D and 3-D US imaging acquisitions were performed using an ultrasonographic system equipped with a single-sweep volumetric transducer (RS80A, CA1-8A transducer; Samsung Medison, Seoul, Republic of Korea).

First, the 2-D US diameter and area were measured on the transverse display, from the leading edge of the adventitia anterior wall to the leading edge of the adventitia posterior wall in peak systole, on-line using a Samsung Medison ultrasonography system (RS80A; Samsung Medison). To obtain a correct anteroposterior image plane on the transverse display, the AAA was confirmed to be horizontal on the longitudinal display (Fig. 1b and c).

Then, the 3-D US acquisition was performed during breath hold (< 2 s), while the transducer was kept in a firm stable position above the cross-section showing Dmax. The image obtained was the axial volume acquisition of the AAA. For the healthy controls, 3-D US images of the field that included the renal arteries and aorta volume distal to them were obtained. Depending on the imaging results, 1–4 acquisitions were performed. The best acquisition was used for the analysis. The 3-D US acquisitions were then transferred to a workstation and later handled in experimental semi-automated 3-D software.

### 3-D US analysis

3-D US acquisitions were analyzed using an experimental automated software (S-3D AAA, Samsung Medison; Fig. 1d). The physicians handled each of their own 3-D US acquisitions. The software automatically detected the aortic wall to generate a centerline and 3-D aorta model. The first step was automatic delineation of the AAA wall, directly in three dimensions. The second step was automatic selection of the centerline, corresponding to the centerline of the AAA walls, not to the centerline of the AAA lumen. The third step was automatic selection of intraluminal thrombus, if present. All three steps were processed simultaneously within 2–3 s. The maximum diameter perpendicular to this centerline on 3-D US was defined as Dmax. Thus, on the orthogonal cross-section, the diameter was not restricted to a specific axis but could be established in any direction. Thrombi were automatically detected using the software. The vessel, lumen, and thrombus areas; vessel volume; and lumen volume were all measured using the same definition used in the CT analysis. Additional manual adjustments were applied for the vessel wall and thrombus-lumen interface, if needed.

### Statistical analyses

Demographic characteristics are reported as percentage or mean ± standard deviation (SD). To test for differences in the observed inter-modality range of variability, and interobserver and intraobserver variabilities, the Pearson correlation coefficient was calculated for the sum and difference of the paired differences made on the same subject. Inter-modality variability was presented using Bland-Altman plots, where the differences between measurements made on the same subject were plotted against the mean outcome, showing the mean difference and the upper and lower limits of agreement given by the mean ± 1.96 **×** SD (2 SD). The intraclass correlation coefficient (ICC) was also calculated to evaluate the inter-modality variability, and intraobserver and interobserver reproducibility. Good correlation was defined as an ICC of > 0.8. The Student paired *t* test was used to compare means and mean differences.

## Results

2-D and 3-D US images were obtained in all the cases (100% technical success). The mean analysis time was 3.0 ± 1.0 s for the generation of an aortic model by automated calculation and 32.4 ± 20.4 s overall, including additional adjustments. Additional adjustments were mainly applied for the thrombus-lumen interface.

Patient characteristics are shown in Table 1. Overall, 77 patients (18 control subjects and 59 patients with AAA) were evaluated. The mean age was 56 ± 12 years in the control group and 72 ± 8 years in the patients with AAA. The mean body mass index was 23.6 ± 3.1 kg/m^2^ in the control group and 24.3 ± 3.2 kg/m^2^ in the patients with AAA.

**Table 1:** Baseline characteristics of the control subjects and patients with abdominal aortic aneurysm

Variable	Overall (*n* = 77)	Control subjects (*n* = 18)	Patients with AAA (*n* = 59)
Age, years	68 ± 11	56 ± 12	72 ± 8
Men, n (%)	61 (79.2)	12 (66.7)	49 (83.1)
Body mass index, kg/m^2^	24.2 ± 3.2	23.6 ± 3.1	24.3 ± 3.2
Systolic blood pressure, mmHg	119.9 ± 11.5	117.0 ± 10.0	120.8 ± 11.8
Diastolic blood pressure, mmHg	73.1 ± 9.6	72.4 ± 9.6	73.3 ± 9.6
Hypertension, n (%)	41 (53.2)	1 (5.6)	40 (67.8)
Diabetes mellitus, n (%)	19 (24.7)	2 (11.1)	17 (28.8)

*AAA* abdominal aortic aneurysm

**Fig. 3:**
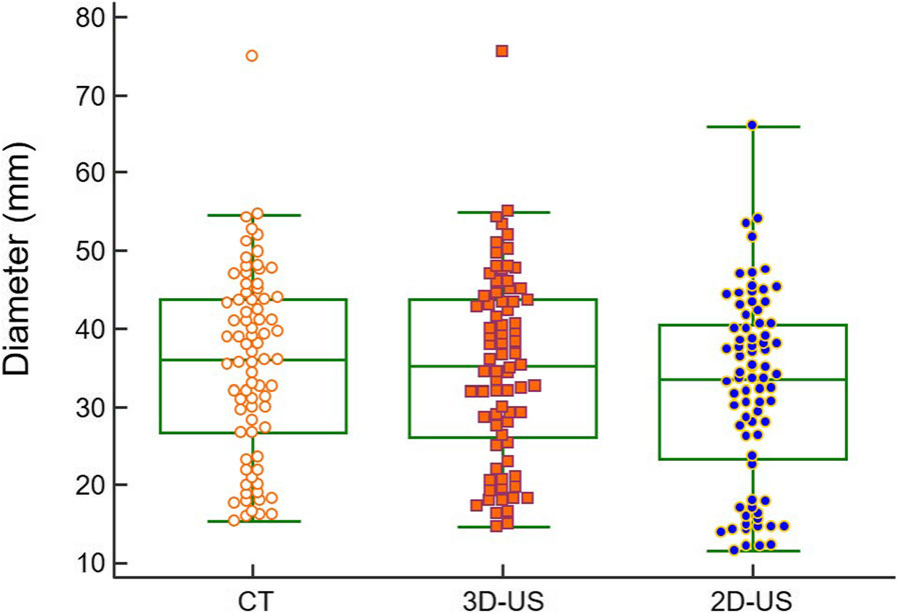
Maximal aortic diameter from each imaging modality. CT, computed tomography; 2-D US, two-dimensional ultrasonography; 3-D US, three-dimensional ultrasonography

Table 2 shows comparisons of the Dmax, vessel area, and vessel volume obtained with 2-D US, 3-D US, and CT. The mean Dmax obtained using CT was 18.9 ± 2.5 mm in the control subjects and 40.1 ± 9.0 mm in the patients with AAA. The mean Dmax obtained using US was smaller than that by CT, and it was 15.0 ± 2.6 mm by 2-D US and 19.0 ± 2.7 mm by 3-D US in the control subjects. The corresponding values were 37.6 ± 8.3 mm in the control subjects and 39.9 ± 9.2 mm in the patients with AAA. The Dmax values of all the patients are shown in Fig. 3.

**Table 2:** Comparison of maximum diameter, vessel area, and vessel volume

Variable	Overall (*n* = 77)	Control subjects (*n* = 18)	Patients with AAA (*n* = 59)
CT			
Dmax, mm	35.4 ± 12.0	18.9 ± 2.5	40.1 ± 9.0
Vessel area, cm^2^	10.9 ± 7.1	2.4 ± 0.6	13.3 ± 6.2
Vessel volume, cm^3^	44.4 ± 36.1	2.1 ± 0.5	57.3 ± 31.4
2-D US			
Dmax, mm	32.3 ± 12.1	15.0 ± 2.6	37.6 ± 8.3
Vessel area, cm^2^	10.3 ± 7.1	1.9 ± 0.4	12.8 ± 6.1
3-D US			
Dmax, mm	35.1 ± 12.0	19.0 ± 2.7	39.9 ± 9.2
Vessel area, cm^2^	10.6 ± 7.0	2.1 ± 0.4	13.1 ± 6.7
Vessel volume, cm^3^	42.4 ± 33.9	1.9 ± 0.4	54.0 ± 29.0

*AAA* abdominal aortic aneurysm, *CT* computed tomography, *Dmax* maximum diameter of the abdominal aorta, *2-D US* two-dimensional ultrasonography, *3-D US* three-dimensional ultrasonography

**Fig. 4:**
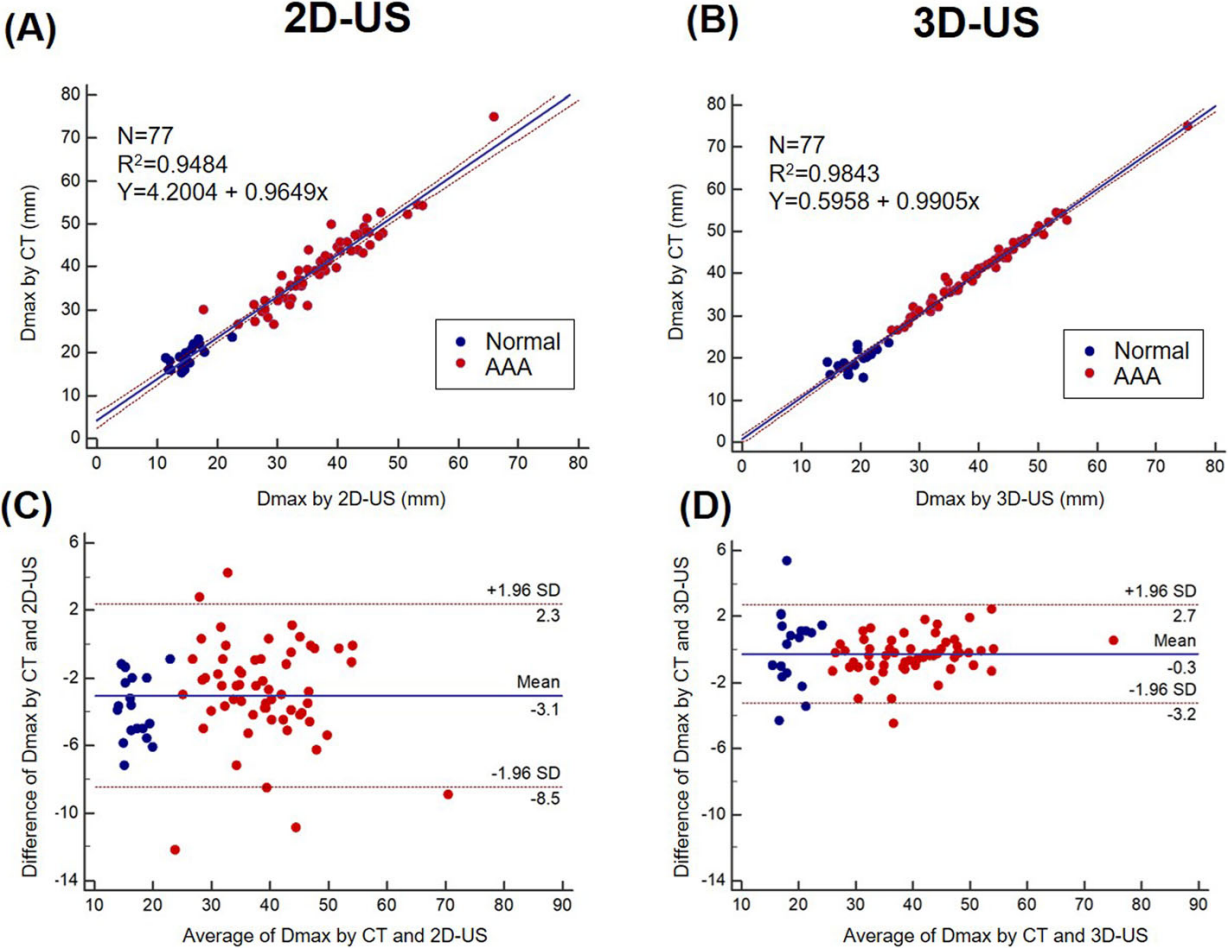
Correlation and agreement between the tests for maximum diameter measurements: (**a**) correlation plot between 2-D US and CT, (**b**) correlation plot between 3-D US and CT, (**c**) Bland-Altman plot between 2-D US and CT, and (**d**) Bland-Altman plot between 3-D US and CT. AAA, abdominal aortic aneurysm; CT, computed tomography; Dmax, maximum diameter of the abdominal aorta; 2-D US, two-dimensional ultrasonography; 3-D US, three-dimensional ultrasonography

Figure 4 shows the correlation and agreement between the tests for Dmax. Overall, the 2-D US methods underestimated Dmax, compared with the 3-D US (32.3 ± 12.1 mm vs. 35.1 ± 12.0 mm; Fig. 4a and b). Figure 4(c and d) shows the Bland-Altman plots for 2-D and 3-D US, compared with CT. The Bland-Altman analysis for 3-D US showed better agreement with CT (2 SD: 2.9 mm) than with 2-D US (2 SD: 5.4 mm).

Table 3 shows the comparisons of the lumen and thrombus measurements in the subgroup of 40 patients with intraluminal thrombus. The thrombus areas were 5.0 ± 5.8 cm^2^ for CT and 5.1 ± 5.2 cm^2^ for 3-D US. The thrombus volumes were 30.6 ± 41.5 cm^3^ for CT and 29.4 ± 37.1 cm^3^ for 3-D US.

**Table 3:** Comparison of lumen and thrombus measurements in the subgroup of 44 patients with intraluminal thrombus

Variable	CT	3-D US
Lumen area, cm^2^	9.0 ± 4.3	8.7 ± 4.2
Thrombus area, cm^2^	5.0 ± 5.8	5.1 ± 5.2
Lumen volume, **c**m^3^	33.8 ± 21.5	31.8 ± 20.8
Thrombus volume, **c**m^3^	30.6 ± 41.5	29.4 ± 37.1

*CT* computed tomography, *3-D US* three-dimensional ultrasonography

Table 4 shows the ICCs between the tests. 3-D US demonstrated better agreement with CT than with 2-D US in Dmax (ICC: 0.992 vs. 0.974), vessel area (ICC: 0.993 vs. 0.980), lumen area (ICC: 0.942 vs. 0.906), and thrombus area (ICC: 0.960 vs. 0.937). Moreover, 3-D US showed good agreement with CT in vessel volume (ICC = 0.995; 95% confidence interval [CI], 0.991–0.997), lumen volume (ICC = 0.967; 95% CI, 0.942–0.981), and thrombus volume (ICC = 0.984; 95% CI, 0.969–0.992).

**Table 4:** Interclass correlation coefficient between ultrasonography and computed tomography

Variable	CT vs. 2-D US	CT vs. 3-D US
	Interclass correlation coefficient (95% CI)	Interclass correlation coefficient (95% CI)
Dmax	0.974 (0.959–0.983)	0.992 (0.988–0.995)
Vessel area	0.980 (0.969–0.987)	0.993 (0.989–0.996)
Lumen area	0.906 (0.830–0.949)	0.942 (0.892–0.969)
Thrombus area	0.937 (0.886–0.966)	0.960 (0.926–0.979)
Vessel volume	–	0.995 (0.991–0.997)
Lumen volume	–	0.967 (0.942–0.981)
Thrombus volume	–	0.984 (0.969–0.992)

*2-D US* two-dimensional ultrasound, *3-D US* three-dimensional ultrasound, *CI* confidence interval, *CT* computed tomography, *Dmax* maximum diameter of the abdominal aorta

**Fig. 5:**
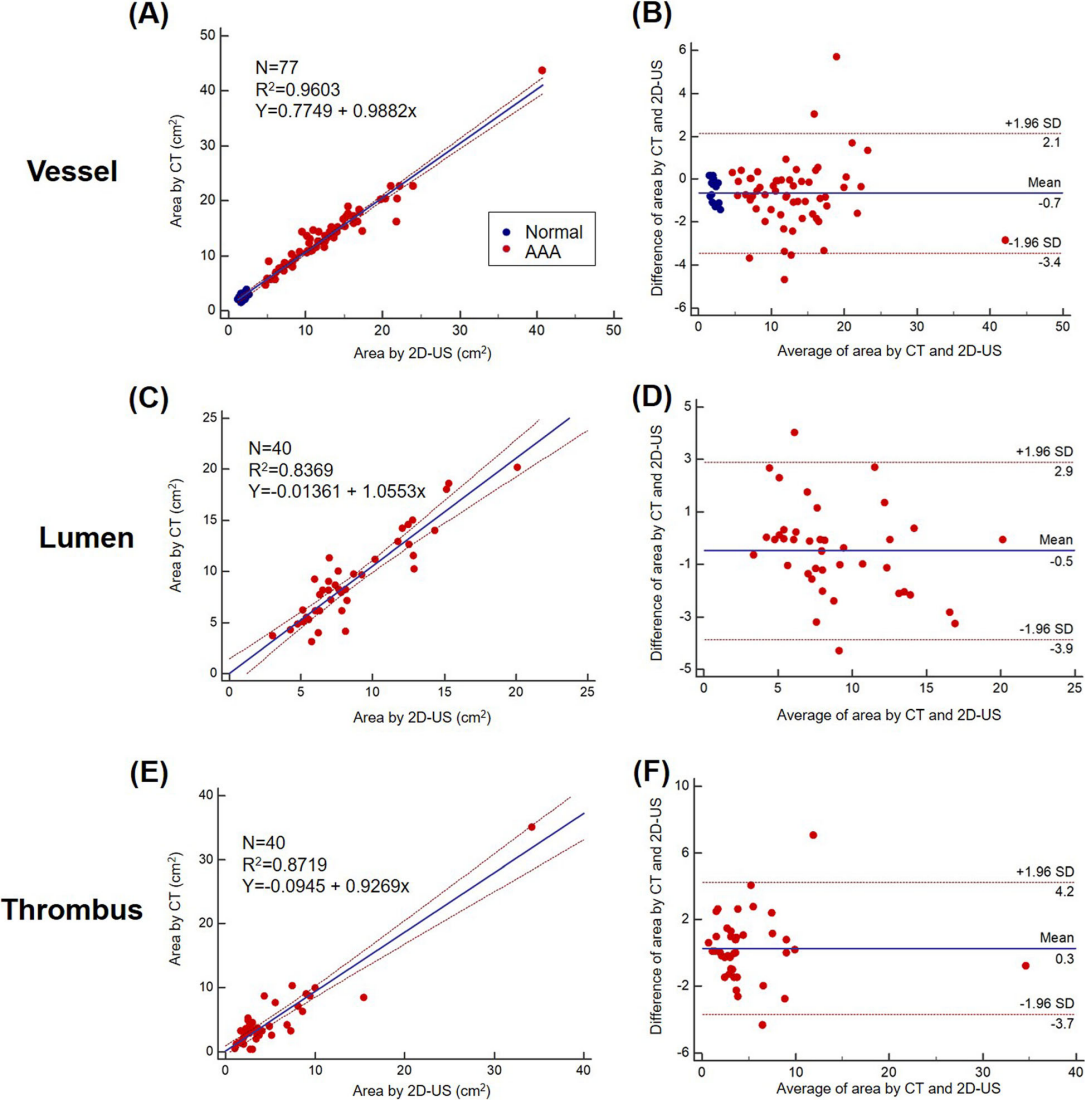
Correlation and agreement between 2-D US and CT for the vessel, lumen, and thrombus areas: (**a**) Correlation plot between the 2-D US and CT values for vessel area, (**b**) Bland-Altman plot between the 2-D US and CT values for vessel area, (**c**) correlation plot between the 2-D US and CT values for lumen area, (**d**) Bland-Altman plot between the 2-D US and CT values for lumen area, (**e**) correlation plot between the 2-D US and CT values for thrombus area, (**f**) Bland-Altman plot between the 2-D US and CT values for thrombus area. CT, computed tomography; 2-D US, two-dimensional ultrasonography; 3-D US, three-dimensional ultrasonography

**Fig. 6:**
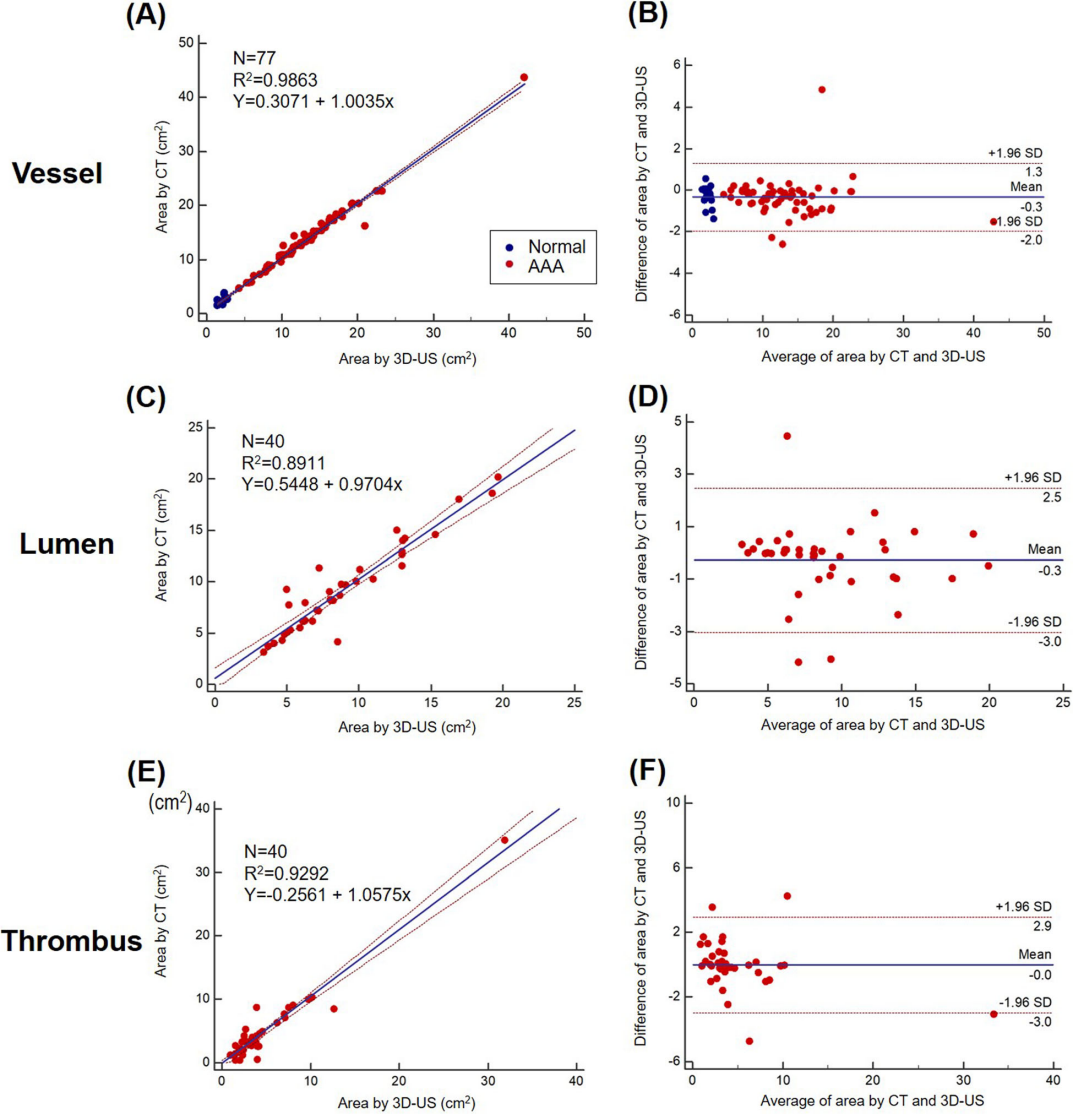
Correlation and agreement between the 3-D US and CT values for the vessel, lumen, and thrombus areas: (**a**) correlation plot between the 2-D US and CT values for vessel area, (**b**) Bland-Altman plot between the 2-D US and CT values for vessel area, (**c**) correlation plot between the 2-D US and CT values for lumen area, (**d**) Bland-Altman plot between 2-D US and CT values for the lumen area, (**e**) correlation plot between the 2-D US and CT values for thrombus area, and (**f**) Bland-Altman plot between the 2-D US and CT values for thrombus area. CT, computed tomography; 2-D US, two-dimensional ultrasonography; 3-D US, three-dimensional ultrasonography

Figures 5 and 6 show the correlation and agreement between the tests for vessel, lumen, and thrombus areas in 2-D US (Fig. 5) and 3-D US (Fig. 6), respectively. Compared with CT, the Bland-Altman analysis for 3-D US showed good agreement than did 2-D US for measurement of vessel area (2 SD: 1.6 cm^2^ vs. 2.8 cm^2^), lumen area (2 SD: 2.8 cm^2^ vs. 3.4 cm^2^), and thrombus area (2 SD: 2.9 cm^2^ vs. 3.9 cm^2^).

**Fig. 7:**
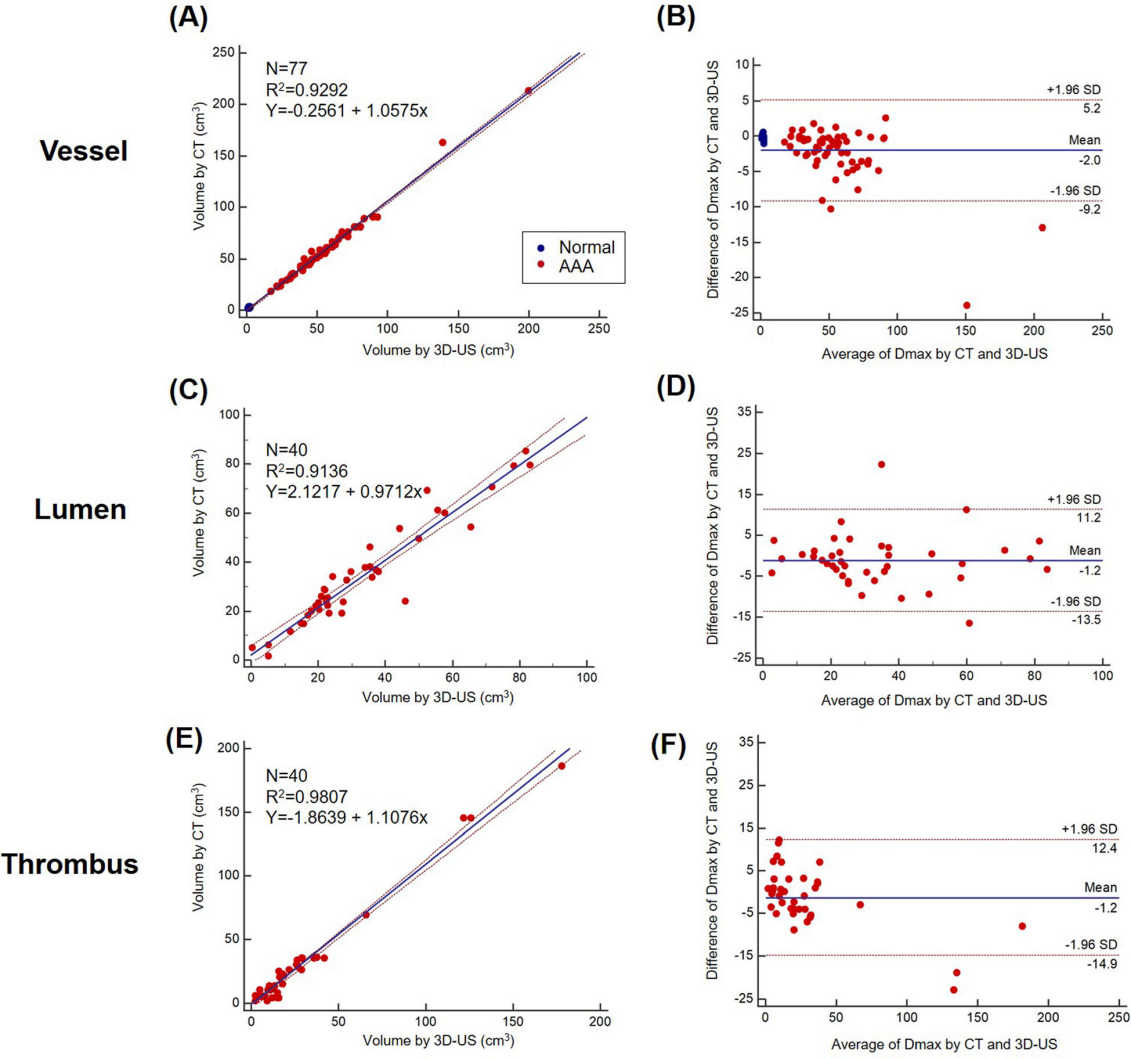
Correlation and agreement between 3-D US and CT values for vessel, lumen, and thrombus volumes: (**a**) correlation plot between the 2-D US and CT values for vessel volume, (**b** and **c**) Bland-Altman plot between the 2-D US and CT values for vessel volume, (**c**) correlation plot between the 2-D US and CT values for lumen volume, (**d**) Bland-Altman plot between the 2-D US and CT values for lumen volume, (**e**) correlation plot between the 2-D US and CT values for thrombus volume, and (**f**) Bland-Altman plot between the 2-D US and CT values for thrombus volume. CT, computed tomography; Dmax, maximum diameter of the abdominal aorta; 3-D US, three-dimensional ultrasonography

Figure 7 shows the correlation (Fig. 7a, c, and e) and agreement (Fig. 7b, d, and f) between 3-D US and CT for volume measurements. The correlation and Bland-Altman analyses for 3-D US showed excellent agreement compared with those for CT for measurement of vessel volume (*R*^2^ = 0.96, 2 SD: 5.7 cm^3^), lumen area (*R*^2^ = 0.91, 2 SD: 12.4 cm^3^), and thrombus area (*R*^2^ = 0.98, 2 SD: 13.6 cm^3^).

**Fig. 8:**
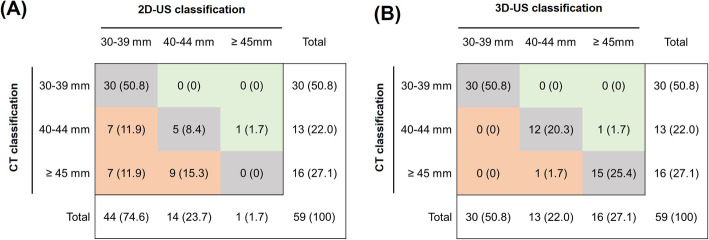
Difference in maximum abdominal aortic aneurysm diameter classification according to the recommended follow-up intervals between 2-D US, 3-D US, and CT: (**a**) 2-D US and CT and (**b**) 3-D US and CT. Data are presented as the number of examinations (%). Gray indicates no reclassification; green, overestimation of risk classification; and red, underestimation of risk classification. CT, computed tomography; Dmax, maximum diameter of the abdominal aorta; 2-D US, two-dimensional ultrasonography; 3-D US, three-dimensional ultrasonography

Figure 8 shows the difference in Dmax classification according to the recommended follow-up intervals by the European Society of Cardiology [9] between 2-D US and CT, and between 3-D US and CT. 3-D US classified 57 patients (96.6%) according to the same categories used in the CT classification, whereas 2-D US classified only 35 patients (59.2%). 2-D US classified 23 patients into the lower risk group (39.0%). Moreover, in 7 patients (11.9%), the risk was underestimated by two grades when 2-D US was used to measure Dmax.

The ICCs for the intraobserver and interobserver reproducibility of Dmax were 1.000 (95% CI, 1.000–1.000) and 1.000 (95% CI, 0.999–1.000), respectively. The ICCs for the intraobserver and interobserver reproducibility of thrombus volume were 0.998 (95% CI, 0.994–0.999) and 0.967 (95% CI, 0.928–0.979), respectively.

## Discussion

Compared with CT, the present study demonstrated that 3-D US was capable of measuring Dmax and vessel area parameters for aortic aneurysm more accurately with better agreement. It can also provide vessel and thrombus volume information with excellent agreement with CT.

### 3-D US for AAA surveillance and risk stratification

Consensus exists that surgery or endovascular repair should be proposed when an AAA reaches a maximum diameter of 55 mm, growing rapidly over 1 cm/year in asymptomatic patients [1]. On the basis of a recent individual-based meta-analysis of trials and observational studies with repeated AAA measurements over time, intervals of 3, 2, and 1 year were proposed for AAAs of 30–39, 40–44, and 45–54 mm in diameter, respectively [9, 16].

2-D US is usually regarded as a first-line study for AAA surveillance. However, it is user dependent, and images should be skillfully acquired after angulation, especially in cases of tortuous aorta. In addition, bias exists toward smaller mean diameter measurements for 2-D US, as it measures the anteroposterior diameter with lower inter-measurement variability [10]. By contrast, measurement of the aorta with 3-D US does not require the assumption of any specific geometry but can be directly computed by manual or automatic segmentation of continuous slice data, which can overcome the limitations of 2-D US and would be the optimal method for AAA surveillance. Therefore, several studies have developed 3-D US-based analyses for AAA [17, 18]; however, until now, no standardized methods for acquisition and analysis have been established.

Here, we propose a novel approach to extract more information from 3-D US acquisitions obtained with a single-sweep volumetric probe by combining the recorded volume with a dedicated post-processing software that makes it possible to extract the surface of the AAA and its centerline. The novelty of this study is that it shows the possibility of automatically extracting the Dmax of the AAA in any direction, perpendicular to the centerline, from the 3-D US AAA segmentation within several seconds. The clinical interest could be to provide a unified definition of Dmax in any direction using 3-D US. Improving the quality of measurements is aimed primarily at improving patient care over the successive stages of the disease, including screening, decision for intervention, and follow-up. Using this 3-D US software, we correctly categorized 96.6% of the patients with the same CT classification for the recommended follow-up intervals, whereas the 2-D US classification matched only in 59.2% of the patients. Therefore, our 3-D analysis system can be used as a first-line modality for AAA screening and surveillance, as it is inexpensive and noninvasive and can screen quickly and easily.

### Assessments of vessel and thrombus volumes

As AAA is a 3-D disease, the clinical interest in AAA volume measurement lies in enabling better prediction of the evolution of small AAAs and of AAAs post endovascular AAA repair. AAA volume estimation has previously been reported with CT acquisitions combined with post-processing [19–22]. Nevertheless, this technique is not routinely used in clinical practice because, compared with US, CT presents drawbacks such as exposure to radiation, injection of iodine contrast medium, and higher costs.

Our novel method automatically calculated the aorta volume. In addition, the novelty of this software is that it automatically quantifies thrombus volume using dedicated software, in addition to the whole vessel volume and area. The impact of intraluminal thrombus on AAA rupture risk is controversial until now. Although several studies have demonstrated that intraluminal thrombus reduces aortic wall stress, thereby acting as a mechanical buffer, [23, 24] increasing evidence shows that high intraluminal thrombus burden may be a surrogate marker of decreased aortic wall strength and a characteristic of high-risk small aneurysms [25, 26]. Our novel software can provide additional information on intraluminal thrombus volume by automatic measurement and therefore facilitate the unraveling of the role of intraluminal thrombus volume that cannot be acquired with 2-D US.

### Limitations

The main limitation of the present study was that because of the probe characteristics, information about the neck was not available for large AAAs. Further development of the hardware and scanning method is expected to facilitate data acquisition and enhance the performance of the system. US imaging of the abdominal aorta may be technically limited because of patient obesity or excessive abdominal gas, even though we did not encounter any technical failure of 3-D US in our study. However, with appropriate patient preparations such as oral intake restriction for at least 8 h before the examination, many of the body habitus pitfalls may be overcome. The mean body mass index of the patients was significantly lower than those of other populations such as US citizens. As obesity decreases the quality of US imaging, our results may not be applicable to other populations with a higher prevalence of obesity. Both CT and US images were acquired without ECG gating in the present study, and changes in AAA according to cardiac cycle were not considered. However, the changes in the abdominal aorta during the cardiac cycle are less significant than those in the thoracic aorta, and abdominal aorta CT is usually performed without ECG gating in routine clinical practice. Our intention was to investigate the accuracy of 3-D US compared with that of CT, and we applied the same protocol to CT and 3-D US without ECG gating. Further studies are warranted to evaluate the clinical value of the cyclic change evaluation.

## Conclusion

Our novel semi-automated 3-D US analysis system provides more accurate Dmax values than 2-D US and provides precise volumetric data, which were not evaluable with 2-D US. The application of semi-automated 3-D US analysis is easy and accurate for abdominal aorta assessment.

## Data Availability

The datasets used and/or analyzed in the present study are available from the corresponding author upon reasonable request.

## Abbreviations

2-D US: Two-dimensional ultrasonography
3-D US: Three-dimensional ultrasonography
AAA: Abdominal aortic aneurysm
CT: Computed tomography
Dmax: Maximum aortic diameter
ECG: Electrocardiography
ICC: Intraclass correlation coefficient
SD: Standard deviation
